# Diaphragmatic hernia as a rare complication of colonoscopy: Case report and literature review

**DOI:** 10.1097/MD.0000000000009660

**Published:** 2018-01-19

**Authors:** Siyu Liu, Mugen Dai, Bin Ye, Zhigang Zhao, Yang Shi, Lirong Peng

**Affiliations:** a Department of Medicine; b Department of Gastroenterology; c Department of Laboratory medicine, The Fifth Affiliated Hospital of Wenzhou Medical University, Lishui, Zhejiang Province, China.

**Keywords:** colonoscopy, complication, diaphragmatic hernia

## Abstract

**Rationale:**

Diaphragmatic Hernia is rare as complication of Colonoscopy. Diaphragmatic hernia as a complication of colonoscopy has been reported only few cases. Additionally, it is often misdiagnosed as other disease by clinicians due to their lack of related knowledge, which delays diagnosis of Diaphragmatic hernia and thus exacerbates the prognosis.

**Patient concerns:**

We report the case of a 66-year-old man with fecal occult blood. In the case, sudden epigastric pain after colonoscopy owing to diaphragmatic hernia in a left hemidiaphragm.

**Diagnoses:**

The diagnoses made by a CT scan without delay. It showed marked protrusion of the large bowel into the left thoracic cavity along with elevation of the left diaphragm.

**Interventions:**

The diaphragmatic defect was repaired by simple closure and intestinal adhesions release surgery.

**Outcomes:**

Five days after surgery, the patient was discharged in good condition.

**Lessons:**

Most of diaphragmatic hernia is congenital with high mortality. However, there are a few cases of Diaphragmatic hernia caused by previous trauma or surgery. We herein report an unusual case of diaphragmatic hernia related to colonoscopy but usually life-threatening complication.

## 1 Introduction

Life-threatening complications of flexible lower gastrointestinal endoscopy are rare. Little is known about the potential danger of unexpected diaphragmatic hernias during diagnostic colonoscopy, and it occurs more frequently on the left than the right side. We herein report a rare case of a patient with a history of esophageal cancer surgery 4 years earlier who underwent colonoscopy for fecal occult blood study positive. Three days later a strangulated loop of bowel was found contained within diaphragmatic hernia in the chest.

## 2 Case presentation

**Figure 1:**
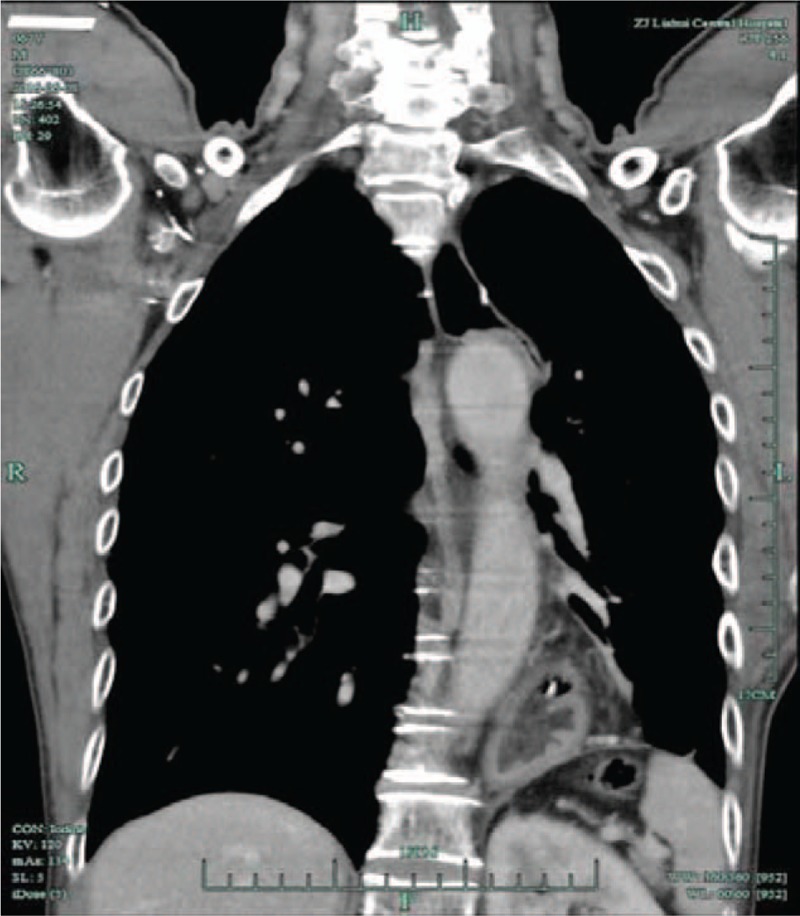
The esophagus and abdominal computed tomography scan is normal before colonoscopy.

A 66-year-old man underwent diagnostic colonoscopy because of fecal occult blood study positive twice. He had history of surgery for esophageal cancer. The esophagus and abdominal computed tomography (CT) scan (Fig. 1) were normal before colonoscopy. Fecal occult blood study was positive. Complete blood count revealed a decreased RBC count (4.35 cells/L) with a borderline low hemoglobin level (127 g/L) and a slightly elevated HCT (red blood cell specific volume) (39.4%).

**Figure 2:**
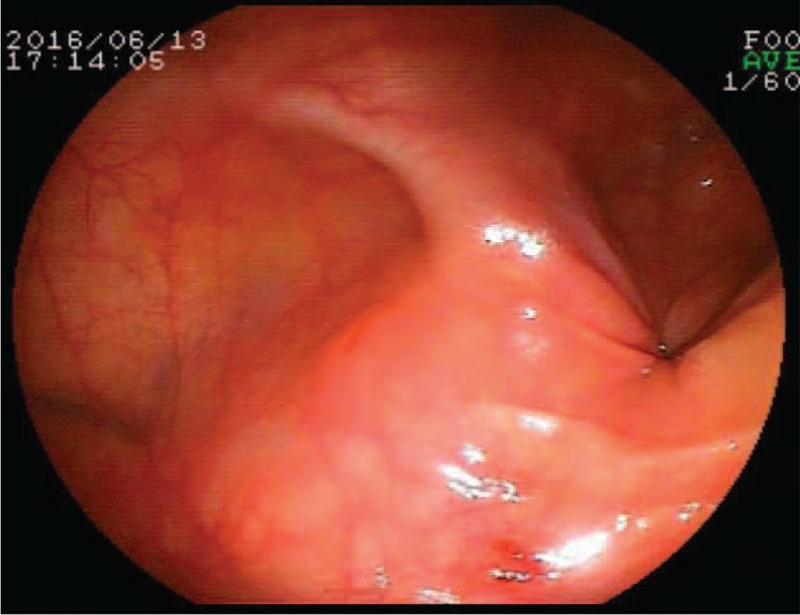
Colonoscopy can be seen descending colon stenosis.

During the colonoscopy process, due to descending colon narrowed (Fig. 2), colonoscopy could not be advanced from the anus 40 cm despite of trying twice. The scope was withdrawn and the injected air was removed.

**Figure 3:**
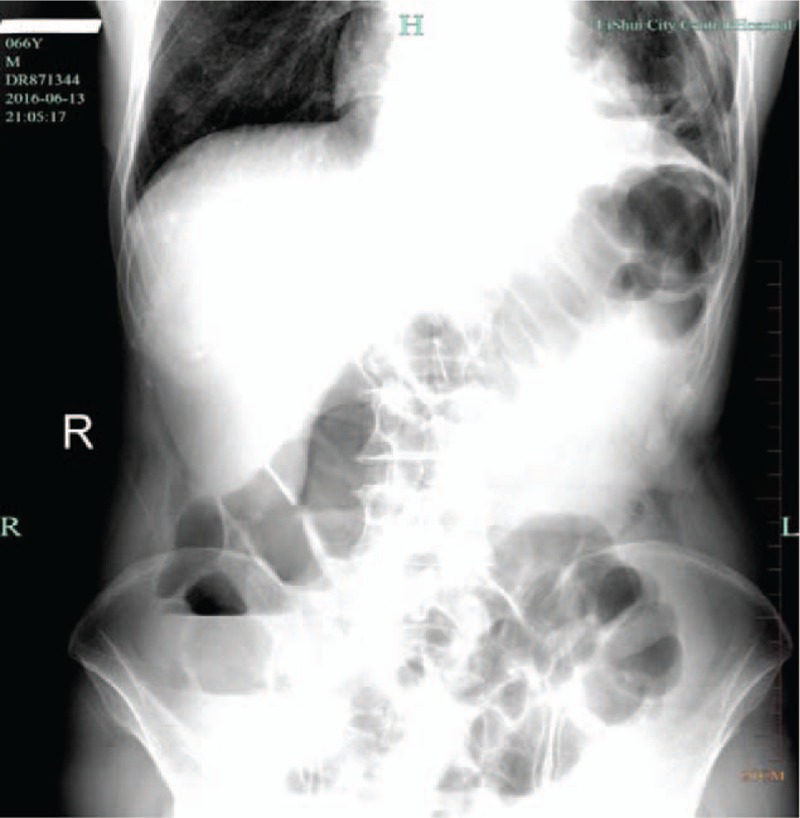
X-rays showed demonstrated several loops of dilated colon within the left upper quadrant.

**Figure 4:**
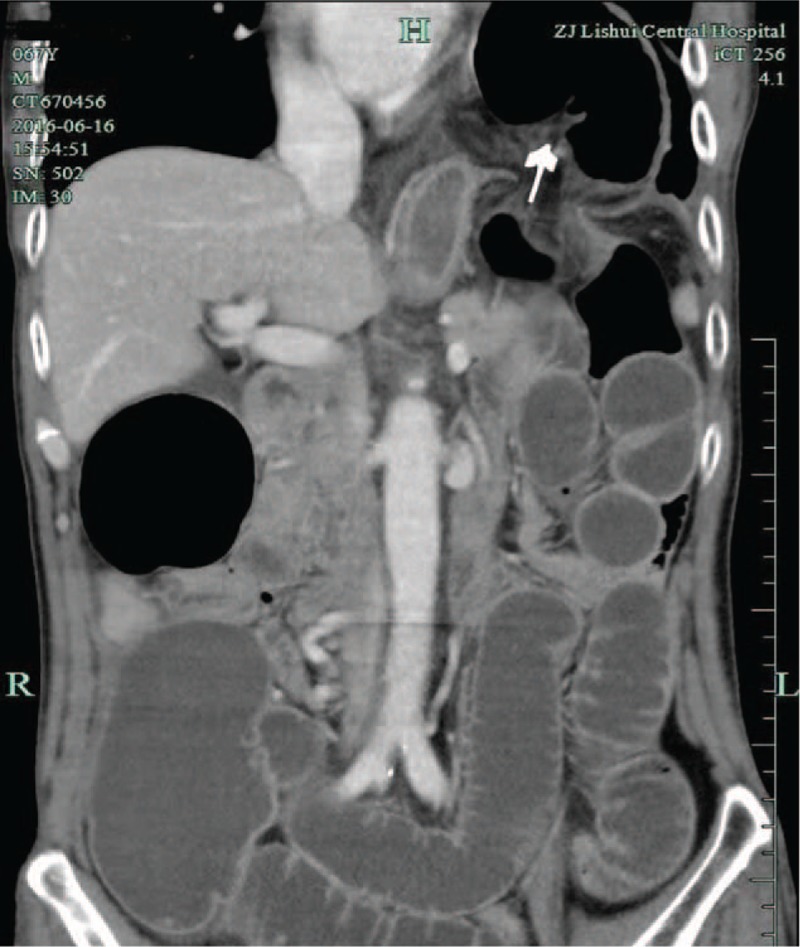
A CT scan of the abdomen was showed marked left diaphragmatic hernia formed (as shown by the arrow). CT = computed tomography.

Within 3 hours of colonoscopy, the patient complaints of abdominal distension, left epigastric pain, no nausea, and no dyspnea. X-rays showed several loops of dilated colon within the left upper quadrant (Fig. 3). A CT scan of the abdomen was showed marked protrusion of the large bowel into the left thoracic cavity along with the elevation of the left diaphragm (Fig. 4). It released diaphragmatic hernia occurred. Laboratory analysis revealed the following after colonoscopy: serum WBC 11.5 cell/L; RBC 4.11 cell/L; Hgb 114 g/L; HCT 35.3%; PLT244K; neutrophils 81.9%; monocytes 10.8%; CRP 71 mg/L; Saa 522.971 mg/L; total protein 48 g/L, pH 7.451; pCO_2_ 36.1 mm Hg; pO_2_ 71.2 mm Hg; HCO_3_ 24.8 mmol/L.

An emergency operation revealed that most of the transverse colon and greater omentum had herniated through a “Y” shape diaphragmatic defect about 6 cm, without any gangrenous or strangulation changes. Abdominal extensive adhesions caused by esophageal cancer postoperative. There was about 300 mL of light red fluid in the abdominal cavity. The diaphragmatic defect was repaired by simple closure and intestinal adhesions release surgery. The bowel was withdrawn into the peritoneal cavity. The patient was discharged in good condition 5 days after the operation.

## 3 Discussion

Diaphragmatic hernia refers to the abdominal organs caused by the weak diaphragm, defect, or traumatic wound into the chest. Clinical divided diaphragmatic hernia into 3 categories: esophageal hiatal hernia, congenital diaphragmatic hernia, and traumatic diaphragmatic hernia.^[1–3]^ Due to herniation into the internal organs of the chest occupy the oppression of lung tissue or heart, mediastinal shift to the contralateral. Patients significantly reduced lung capacity, resulting in shortness of breath and difficulty in breathing, Cyanosis occurs in severe cases, cardiac displacement of the blood flows to the venous obstruction, reduced cardiac output, causing rapid heart rate, blood pressure, and even leads to shock state. Some serious patients can cause toxic shock.^[4]^ Colonoscopy is a remarkable safe and effective method of diagnosing colon disease, which is widely used in clinical.^[5]^ Most of the complications can be avoided by complying with general endoscopic rules, such as adequate and clean gut preparation and paying attention to contraindications.^[6]^ In recent years, various techniques of single-operation colonoscopy have been rapidly developed; further alleviating the suffering and complications of the subjects. Although the complications of colonoscopy are rare, but inevitable, it is reported that the incidence is generally below 0.35%.^[7]^ Little is known about diaphragmatic hernias as a possible risk during colonoscopy. Because of clinical rare, the incidence of diaphragmatic hernia caused or exacerbated by diagnostic colonoscopy has not yet been fully elucidated, and has not caused the full attention of endoscopic physician.^[4,8,9]^ Therefore, prior to colonoscopy, various risk factors should be assessed to avoid complications and to facilitate the early diagnosis of diaphragmatic hernia at colonoscopy.

Since the establishment of our hospital, this is the first case of diaphragmatic hernia induced by colonoscopy. In this case, patients have esophageal cancer surgery history. Emergency laparotomy, showing esophageal cancer changes after surgery, abdominal adhesions. The patients with diaphragmatic hernia are mainly due to the following reasons^[10]^: esophageal cancer may have a potential risk of diaphragmatic weakening or deformity; intestinal cavity caused by extensive adhesions stenosis; endoscopic physician forced too much or too fast lead to increased intra-abdominal pressure, resulting in difficulty into the mirror and inject more air, so aggravate the diaphragm weakness or defect damage. Therefore, intra-abdominal contents easily pass through the weak diaphragm or previous diaphragm defect into the chest, thus diaphragmatic hernia occur.

To our knowledge only few cases of diaphragmatic hernia as a complication of colonoscopy occur. Only seven cases were reported previously, during 1995 to 2016. Similar cases of this article occurred in China in1995. The patient had also esophageal cancer surgery history. Diaphragmatic hernia occurred when the front of the colonoscopy passes through the spleen, ileus occurred, not perforated, recovery after emergency surgery. The remaining 6 patients are elderly, 3 of them had history of previous abdominal trauma surgery, 1 had intestinal perforation, 1 was positive for faecal occult blood, and 1 was respiratory distress. Therefore, patients with a history of trauma, especially splenectomy, in addition to attach importance to the complications of colonoscopy, but also should be alert to postoperative complications.^[4,11–14]^

For patients who have a history of abdominal surgery or who have had a traffic accident, blunt injury, or any other condition that may impair the history of diaphragmatic injury, endoscopy physicians should give more attention during colonoscopy. Before colonoscopy, asking in detail whether there is a corresponding history of complications that may occur, especially the history of abdominal trauma surgery. Pay close attention to the patients’ reaction of the colonoscopy, and communicate timely to understand the patients’ feeling. The examination should be stopped timely if it is difficult to keep operation. And it is forbidden to enforce the operation. Observe closely if there is complication after the examination.

## 4 Conclusion

In summary, it is rarely to diaphragmatic hernia as adverse complication of colonoscopy, and serious consequences can be life threatening. Patients with the corresponding clinical symptoms during colonoscopy should first select chest x-rays and CT to confirm diaphragmatic hernia, and most patients require surgery.
